# Antiplasmodial Activity of Hydroalcoholic Extract from Jucá (*Libidibia ferrea*) Pods

**DOI:** 10.3390/pharmaceutics15041162

**Published:** 2023-04-06

**Authors:** Francisco Flávio Vieira de Assis, José Sousa de Almeida Junior, Tânia Mara Pires Moraes, Fernando de Pilla Varotti, Camila Castilho Moraes, Adilson Sartoratto, Waldiney Pires Moraes, Antonio Humberto Hamad Minervino

**Affiliations:** 1 Laboratory of Animal Health, LARSANA, Federal University of Western Pará, UFOPA, Santarém 68040-255, Brazil; 2 Laboratório de Farmacologia Experimental, Universidade Federal do Oeste do Pará, UFOPA, Santarém 68040-255, Brazil; 3 Núcleo de Pesquisa em Química Biológica (NQBio), Universidade Federal de São João Del Rei, Campus Centro-Oeste, Av. Sebastião G. Coelho, 400, Chanadour, Divinópolis 35501-296, Brazil; 4 Centro Pluridisciplinar de Pesquisas Químicas, Biológicas e Agrícolas, Universidade de Campinas—UNICAMP, Campinas 13148-218, Brazil

**Keywords:** malaria, Jucá, herbal medicines, Amazon

## Abstract

Malaria is an infectious and parasitic disease caused by protozoa of the genus *Plasmodium*, which affects millions of people in tropical and subtropical areas. Recently, there have been multiple reports of drug resistance in *Plasmodium* populations, leading to the search for potential new active compounds against the parasite. Thus, we aimed to evaluate the in vitro antiplasmodial activity and cytotoxicity of the hydroalcoholic extract of Jucá (*Libidibia ferrea)* in serial concentrations. Jucá was used in the form of a freeze-dried hydroalcoholic extract. For the cytotoxicity assay, the(3-[4,5-dimethylthiazol-2-yl]-2,5 diphenyl tetrazolium bromide (MTT) method with the WI-26VA4 human cell line was used. For the antiplasmodial activity, *Plasmodium falciparum* synchronized cultures were treated with serial concentrations (0.2 to 50 μg/mL) of the Jucá extract. In terms of the chemical composition of the Jucá extract, gas chromatography coupled to mass spectrometry measurements revealed the main compounds as ellagic acid, valoneic acid dilactone, gallotannin, and gallic acid. The Jucá hydroalcoholic extract did not show cytotoxic activity per MTT, with an IC_50_ value greater than 100 µg/mL. Regarding the antiplasmodial activity, the Jucá extract presented an IC_50_ of 11.10 µg/mL with a selective index of nine. Because of its antiplasmodial activity at the tested concentrations and low toxicity, the Jucá extract is presented as a candidate for herbal medicine in the treatment of malaria. To the best of our knowledge, this is the first report of antiplasmodial activity in Jucá.

## 1. Introduction

Malaria, an infectious and parasitic disease, has a wide distribution in tropical and underdeveloped regions, including countries in South America such as Brazil, and affects millions of people [1]. According to the World Malaria Report [2], in 2021, there were an estimated 247 million malaria cases in 84 countries with 619,000 deaths. The Amazon region concentrates most cases of malaria in Brazil, which can be attributed to several factors, including favorable conditions for the development of the vector and inadequate sanitation conditions [1,3].

Protozoa belonging to the *Plasmodium* genus are the causative agents of malaria and are transmitted to humans by infected female of *Anopheles* mosquitoes. With its impact felt worldwide, *Plasmodium* is the protozoan responsible for putting over 2.4 billion people (roughly 40% of the global population) at risk of malaria in more than 100 countries [4]. *Plasmodium falciparum* is the most dangerous species because it can transmit cerebral malaria, the most severe form of the disease that, in most cases, leads to death [5]. The artemisinin-derivative resistance in southeast Asia observed in *P. falciparum* [6] and, more recently, artemisinin partial resistance in Africa [2] threaten malaria control, increasing the need for new drugs for malaria treatment [3].

Plants have greater diversity in tropical climates, and traditional medicine based on herbal preparations is commonly used in developing countries, especially in the Amazon region [7]. Natural-substance-derived medicines are capable of treating approximately 80% of all the diseases that affect humanity. These medicines exhibit various pharmacological activities such as antimicrobial, anti-inflammatory, antiproliferative, anticoagulant, antiparasitic, and immunosuppressant activities, among others [8,9,10].

The significance of natural products, specifically those derived from plants, in the development of modern therapeutic drugs is widely acknowledged. It is estimated that around 25% of the drugs currently available were developed from medicinal plants [11,12]. The Brazilian Amazon houses an extensive array of plant species, which hold tremendous potential for the discovery of new secondary metabolites with antiplasmodial activity [3].

*Libidibia ferrea* Mart. Ex tul. Var. ferrea is a tree species found throughout Brazil, popularly known as Jucá [13,14]. In the Amazon region, Jucá (*L. ferrea*) is widely used in popular medicine to treat various health conditions and, in the form of infusions, can be used for the treatment of bronchopulmonary conditions, diabetes, rheumatism, cancer, and diarrhea; however, the most common use is as an alcoholic solution with Jucá pods for the topical treatment of wounds. There are also reports of its antiprotozoal activity [15,16,17].

Extensive research has been conducted on Jucá, focusing on its diverse biological properties, including anti-inflammatory, analgesic, anticancer, antioxidant, antiulcer, and antimicrobial activities [16,18,19,20,21,22,23]. Phytochemical studies of Jucá have revealed the presence of phenolic compounds, tannins, and flavonoids [22,24].

In this context, we aimed to carry out the first study of the in vitro antiplasmodial activity of the hydroalcoholic extract of Jucá on the W2 strain of *P. falciparum*, chemically characterize its constituents, and evaluate its cytotoxicity.

## 2. Materials and Methods

### 2.1. Botanical Material

Jucá was used as a hydroalcoholic extract of its pods. As a matrix, an adult specimen of the species Jucá located in the urban area of Santarém, Pará, was used, which has already been identified and cataloged as FABACEAE—*Libidibia ferrea* (exsiccate HSTM010436, HSTM/UFOPA herbarium). The Jucá hydroalcoholic extract was prepared as reported elsewhere [25]. Briefly, the Jucá pods (1.5 kg) were collected from an adult specimen, cleaned with 70% alcohol, and left at room temperature for the first drying. After 48 h, the fruits were placed in a microprocessing forced air circulation oven at 40 °C for a period of 72 h for drying. Then, the pods were crushed in a knife mill. The crushed material was placed for maceration in alcohol (96%), in the proportion of five liters of alcohol to one kilogram of plant material, for seven days. Then, the macerate was filtered, and the tincture was extracted using a rotary evaporator. The obtained extract was then lyophilized and frozen until its use in the analysis.

### 2.2. Chromatographic Analysis

The analysis of the chemical composition of the ethanolic extract of Jucá pods was performed with a gas chromatograph coupled to an mass spectrometer (model HP-6890, Agilent Technologies, Santa Clara, CA, USA) equipped with a selective mass detector and an HP-5MS capillary column (30 m × 0.25 mm × 0.25 μm), using helium as carrier gas (1 mL/min) under the following conditions: injector = 220 °C, column = 60 °C, heating rate = 3 °C/min up to 280 °C (20 min), and detector = 250 °C. The mass spectra obtained were compared with those in the electronic library of the equipment (NIST-05) and in the study by Adams [26].

### 2.3. Solubilization of the Compound for Tests of Biological Activity

For the preparation of the stock solution, the solvent dimethyl sulfoxide (DMSO) was used, resulting in an initial concentration of 10,000 µg/mL. This solution was kept in a refrigerator at approximately 4 °C. On the day the cytotoxicity tests were performed, dilutions were performed using RPMI, resulting in the following concentrations from the stock solution: 1000, 100, 10, 1, and 0.1 μg/mL. From these concentrations, serial dilutions were performed to achieve 100, 10, 1, 0.1, and 0.01 μg/mL final concentrations of the compounds in the cell plates. The final volume percentage of the DMSO concentration was 0.01%.

### 2.4. Cultivation of Human Cell Lines

For the toxicity analysis, we used the human cell line WI-26VA4 (ATCC CCL-75 lung fibroblasts) obtained from the animal cell bank of the Cell Biology Service (SBC) at the Ezequiel Dias Foundation (FUNED) in Belo Horizonte, MG, which has a laboratory with an ABNT NBR ISO9001/2008 certification and stands out in supporting the development of research projects that require access to cell and tissue culture techniques and in consolidating itself as a reference in the cultivation of animal cells.

The cells were cultivated from a cryopreserved ampoule, as described elsewhere [26,27]. Briefly, cells were thawed at 37 °C, transferred to tubes with RPMI 1640 medium, and centrifuged at 240× *g* for five minutes. The pellet was resuspended in RPMI 1640 medium and 10% heat-inactivated fetal bovine serum. The cells were transferred to culture bottles and maintained as monolayers (37 °C and 5% CO_2_). Bottles were observed for cell morphology and monolayer formation. The freezing of these cells was carried out in cryopreservation ampoules with a solution containing RPMI medium complete with 5% DMSO, and these were kept in liquid nitrogen in the cryopreservation bank (CryoPlus 7405, Thermo Scientific, Waltham, MA, USA). After reaching 80% culture confluence in the T75 bottle, the cells were picked or used in cytotoxicity assays [27,28].

### 2.5. Cytotoxicity Assays

The most used chemosensitivity test in preclinical practice is the MTT test, which refers to the acronym of the reagent used in the final evaluation, that is, 3-(4,5-dimethylthiazol-2-yl)-2,5 diphenyl tetrazolium bromide [29]. The methodology for the MTT analysis is described elsewhere [30,31,32].

The absorbance per well was measured at a wavelength of 550 nm using Gen5 (Data Analysis Software, Bio-Tek). The data were analyzed from distinct experiments. The minimum lethal dose that inhibits the growth of cells in the presence of the test compound by 50% (IC_50_) was determined in comparison with cells cultured without the compound (considered as 100% growth). Calculations were performed with OriginPro Software version 8.0 (OriginLab Corporation, Northampton, MA, USA) using sigmoidal dose–response concentration curves [27,28].

### 2.6. Assessment of Hemolytic Activity

The hemolytic activity was measured as previously described [33]. Briefly, fresh ethylenediaminetetraacetic acid (EDTA)-containing blood was centrifuged, and a red blood cell (RBC) pellet was washed then resuspended using saline (0.9%) sterile solution to obtain a 2% (*v*/*v*) red blood cell solution. The test compound was diluted in a 1% DMSO solution in 8 serial dilutions from 2000 to 15.6 μg/mL and tested in triplicate, and added to the 2% red blood cell suspension. After incubation and subsequent centrifugation, the hemoglobin release was measured by absorbance (Abs) at 450 nm using a BioTek Synergy HT multiplate reader. Positive (red blood cells with Triton X-100 1%) and negative (0.9% saline) controls were used.

The percentage of hemolysis was determined with the following equation [34]:

$$\%\;hemolysis=\frac{Abs450\;nm\;sample\;treated\;–Abs450\;nm\;untreated}{Abs450\;nm\;Positive\;control\;–Abs450\;nm\;untreated}\times 100$$

### 2.7. In Vitro Culture of Plasmodium *spp.*

Currently, the only species capable of being continuously cultivated in vitro is *P. falciparum* because it has the capacity to invade young and mature erythrocytes [35]. *P. vivax* only invades reticulocytes (young erythrocytes), which is one of the reasons a continuous culture is not satisfactory [36]. Therefore, the most used model in the screening of new compounds with antiplasmodial action focuses on *P. falciparum* in in vitro tests [37].

### 2.8. In Vitro Culture of Intraerythrocytic Stages of Plasmodium falciparum

Chloroquine-resistant parasites (W2 strain) were cultivated in human red blood cells in vitro under previously established conditions [35]. Parasites were grown in culture bottles with 5% hematocrit using a complete culture medium (RPMI 1640 supplemented with 25 mM HEPES, 21 mM sodium bicarbonate, 300 μM hypoxanthine, 11 mM glucose, 40 μg/mL gentamicin, and 10% (*v*/*v*) heat-inactivated human plasma). The plates were kept at 37 °C, an adequate oxygen concentration was obtained by burning a candle, and daily medium changes were performed. Parasitemia was monitored daily in Giemsa-stained smears under an optical microscope (1000×).

### 2.9. Determination of Parasitemia

Parasitemia was determined as described in a previous report [33]. Briefly, cultured blood smears were made, air-dried, fixed with methanol, and stained with freshly diluted Giemsa solution at a rate of three drops for each 1 mL of buffered saline solution at pH 6.8. After 10 min, the slides were washed under running water, air-dried, and examined with an optical microscope equipped with an immersion lens (100×). Parasitemia was determined by counting the number of infected red blood cells. When the parasitemia rate was greater than 5%, 1000 RBC were counted; however, in the case of very low infection rates (<5%), 6000 cells were counted, and the evaluation was performed by estimating the total number of red blood cells per microscopic field in a total of 50 to 100 fields to estimate the number of infected red blood cells. Parasitemia is expressed as a percentage of parasitized red blood cells.

### 2.10. Synchronization of Plasmodium falciparum Cultivation

The cultured parasites were synchronized using the sorbitol method [38]. Cultures with a predominance of young forms (rings), obtained shortly after synchronization, were used in the chemotherapy trials. After the addition of 10 mL of sorbitol, the parasites were kept under the same environmental conditions as those in the culture (37 °C and 5% CO_2_) for 10 min. After the sorbitol action time, the contents were centrifuged in a falcon tube at 1050× *g* for 5 min, and the volume of the sediment was used to determine the hematocrit. The supernatant fluid was removed, and the red blood cells were resuspended in RPMI culture medium with the hematocrit adjusted to 2%.

### 2.11. In Vitro Schizonticidal Testing with Plasmodium falciparum

In vitro schizonticidal testing with the SybrGreen I antimalarial assay was performed using synchronized cultures of *P. falciparum* with 0.5% ring-stage parasitemia and 2% hematocrit distributed in a 96-well plate (180 μL per well) [39]. The test compound was added (20 μL) to the test plate in triplicate and at different serial concentrations from 50 to 0.20 μg/mL. Control wells contained infected red blood cells without the addition of the test compound (negative control). The standard antimalarial, chloroquine, was tested in parallel in all experiments, at serial dilutions from 500 to 2.0 ng/mL (positive control). In six wells, 180 µL of nonparasitized erythrocytes was added to exclude their autofluorescence.

The test plates were incubated at 37 °C for 48 h. After incubation, the supernatant was removed, and 150 µL of 1X PBS was added to each well. The plates were centrifuged at 700× *g* for five minutes, the supernatant was again removed, and 120 µL of lysis buffer with Sybrsafe (20 mM TRISbase, 5 mM EDTA, 0.008% *w*/*v* saponin, 0.08% *v*/*v* Triton X-100, and 0.2 µL/mL Sybrsafe) was added.

After lysing the erythrocytes, the wells were homogenized, and 100 µL of the contents of each well was added to a new plate containing 100 µL of PBS. The fluorescence reading was performed after incubation for 30 min protected from light in a fluorimeter with excitation of 484 nm and emission of 535 nm.

Parasitized red blood cells (iRBCs) emitted greater fluorescence than normal red blood cells (RBC), with the response of the test compounds being inversely proportional to the fluorescence emission, compared with wells without the addition of the compound.

### 2.12. Selectivity Index

An important criterion in the search for an active compound with therapeutic potential is to determine the absence of toxic effects on host cells through the selectivity index (SI), which measures how much the compound is active against the parasite without causing damage to cell viability in mammals. Thus, the greater the proportion, the greater the selectivity of the compound for parasite cells. The selectivity index was calculated according to the formula below.

$$SI=\frac{IC50\;of\;compound\;in\;mammalian\;cell\;line}{IC50\;of\;the\;compound\;in\;parasitic\;lineage}$$

### 2.13. Statistical Analysis

Statistical analysis was performed using the GraphPad Prism 5.0 program (Prim software, Irvine, CA, USA). The results are expressed as mean +/− standard deviation. Analysis of variance (two-way ANOVA) was applied to assess the statistical significance of differences between study groups. A *p*-value < 0.05 was considered as a criterion for statistical significance.

## 3. Results

### 3.1. Chromatographic Analysis

**Figure 1:**
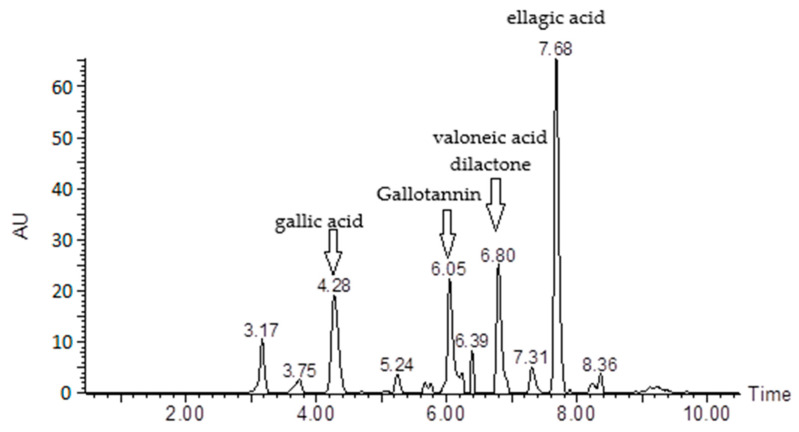
HPLC-DAD chromatogram of hydroalcoholic extract of Jucá. AU: absorbance. Numbers within the compound name indicate the retention time (min).

The phytochemical study of the hydroalcoholic extract of Jucá resulted in the identification of the following compounds: ellagic acid (retention time (rt) 7.68 min), valoneic acid dilactone (rt 6.8 min), gallotannin (rt 6.05 min), and gallic acid (rt 4.28 min) (Figure 1).

The quantification of the compounds identified 78.1% of the constituents in the sample, with ellagic acid being the major compound at 34.27% (Table 1).

### 3.2. Antiplasmodial Activity and Cytotoxicity

The hydroalcoholic extract of Jucá exhibited no cytotoxic activity at concentrations greater than 100 µg/mL. However, the antiplasmodial test determined the IC_50_ value to be 11.10 µg/mL. Moreover, the selectivity index (SI), which represents the ratio between the cytotoxic and antiplasmodial activities of the extract, was also determined (Table 2).

### 3.3. Hemolytic Activity

**Figure 2:**
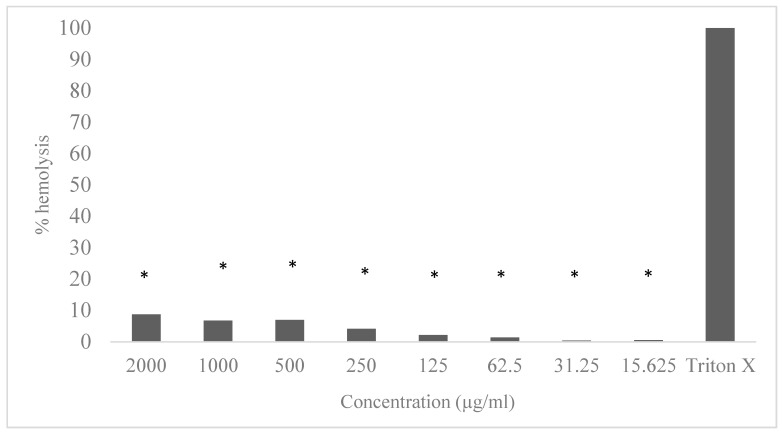
Hemolytic capacity of Jucá extract at different concentrations. * Statistically significant differences in relation to positive control Triton X (*p* < 0.05).

The Jucá extract presented a hemolysis of 8.68% at a concentration of 2000 µg/mL, 6.71% at a concentration of 1000 µg/mL, 6.97% at a concentration of 500 µg/mL, 4.07% at a concentration of 250 µg/mL, 2.13% at a concentration of 125 µg/mL, 1.34% at a concentration of 62.5 µg/mL, and 0% at concentrations of 31.25 µg/mL and 15.62 µg/mL. The data are displayed in Figure 2. All the concentrations showed a difference compared with the positive control group (*p* < 0.05).

## 4. Discussion

GC-MS (gas chromatography–mass spectrometry) showed that the major compounds were gallic acid, gallotannin, valoneic acid dilactone, and ellagic acid. All identified compounds belong to the group of tannins. Américo et al. [25], using thin-layer chromatography, identified a marked presence of hydrolyzable tannins and flavonoids in a *L. ferrea* extract. Studies have indicated that these phenolic compounds have low toxicities and various biological activities, such as in the treatment of cancer, Alzheimer’s disease, neuroinflammation, gastrointestinal problems, inflammation, and leishmaniasis [40,41,42,43,44,45,46]. Previous reports have shown a wide range of antiplasmodial activities in tannin-rich plants [47,48,49]. Lutgen [50] presented an interesting argument regarding the importance of tannins in the prophylaxis of malaria and mentioned the presence of such substances at high concentrations in *Artemisia* plants, but this hypothesis needs further investigation.

In vitro cultivation of *P. falciparum* strains has been widely used to initially screen for potential new compounds because of its low cost and relative simplicity compared with in vivo evaluation [51]. Using this method, we found that the hydroalcoholic extract of Jucá presented moderate antiplasmodial activity, with an IC_50_ of 11.10 µg/mL against chloroquine-resistant *P. falciparum*. Working with a different Amazonian plant, Assis et al. [33] obtained an IC_50_ value of 1.21 µg/mL for the *P. falciparum* W2 strain using a *Cyperus articulatus* residue extract. In another study on the same plant (*C. articulatus*) but using the essential oil, a similar IC_50_ was obtained [52]. This may indicate an unraveled potential that Amazon biodiversity poses to the discovery of new antiplasmodial compounds.

The tannin-rich fractions and ellagitannins in *Punica granatum* did not inhibit the growth of *P. falciparum* [47]; however, some isolated compounds (punicalagins and gallagic acid) in this plant did show activity, with IC_50_ values from 7.5 to 10.9 µg/mL against *P. falciparum* D6 and W2 strains [47]. The in vitro test was adequate for the initial screening but a follow-up in vivo test in a mouse model is required to confirm the antiplasmodial activity of the plant formulations. A study on Nigerian plants showed the chemosuppression of malaria parasites at up to 73% [49].

In addition to antiplasmodial activities, it is important that tested products, especially herbal formulations, are safe and have limited side effects and low toxicities. Chloroquine, one of the most used antiplasmodial drugs, can produce toxicity related to the cardiovascular, hepatic, and ocular systems [53]. The hydroalcoholic extract of Jucá did not present cytotoxic activity in the cell viability assay with MTT and had a hemolysis of <9% at a maximum concentration of 2000 µg/mL, with 0% hemolysis at concentrations below 31.25 µg/mL. Similar results were obtained for *C. articulatus* essential oil, which showed a noncytotoxic effect for human lung fibroblasts [52].

The observed hemolytic activity is considered to be between low and medium. Moreno et al. [54], evaluating the hemolytic activity of different plant extracts, concluded that the degree of hemolysis was low for plantain and burdock (5%) and medium for horsetail, guaco, and blackberry (25%) at the tested concentrations. Pasquini-Neto et al. [55], working with *Pterogyne nitens* leaf extract, classified hemolysis of up to 10% as low hemolytic action. A study on plant extracts, essential oils, and hydrolates from *Zingiber officinale* and *Allium sativum* plants considered low hemolytic activity as being up to 5.56% hemolysis [56]. These findings corroborate the classification of the hemolytic activity described in this study.

The hydroalcoholic extract of Jucá did not present cytotoxicity and had low hemolytic activity. Despite the low selectivity index, the extract showed relevant antiplasmodial activity and can be further examined to determine which compound may be related to this biological activity.

Currently, the *P. falciparum* from the Greater Mekong subregion has developed resistance to most of the commonly used antimalarials [57]. In recent years, the emergence and spread of *Plasmodium* populations resistant to artemisinin have increased the rate of failure in artemisinin-based malaria treatments [58]. Currently, artemisinin-based dual- or triple-combination therapies are showing satisfactory efficacy. Nonetheless, there is an urgent need for alternative treatments and orally administered drugs with new mechanisms of action to effectively combat the malaria parasite [59,60]. Faced with this need, traditionally used Amazonian plants are important for the discovery of antimalarials against *P. falciparum* and *P. vivax* [61].

## 5. Conclusions

The hydroalcoholic extract of Jucá showed satisfactory antiplasmodial activity against the tested W2 strain and was found to be safe in consequence of its low cytotoxicity and hemolytic activity. To the best of our knowledge, this is the first report of antiplasmodial activity in Jucá, a widely used plant in Amazon traditional medicine; therefore, there is a need for deepening and continuing the investigation of the antiplasmodial activity of Jucá, extending to analyzes of isolated compounds and in vivo activity, as well as to other *Plasmodium* species.

## Figures and Tables

**Table 1:** Chemical composition of hydroalcoholic extract of Jucá.

rt (min)	Compound	Rel.%
4.28	Gallic acid	14.75
6.05	Gallotannin	15.19
6.8	Valoneic acid dilactone	13.89
7.68	Ellagic acid	34.27
TOTAL:		78.1

rt: retention time; Rel.%: relative percentage (fraction, in percentage, of the total integrated area for the chromatogram).

**Table 2:** In vitro results for antiplasmodial activity (IC_50_) of Jucá hydroalcoholic extract against *P. falciparum* strain W2, IC_50_ in human cell line WI-26-VA-4, and extract selectivity index.

Test Substance	IC_50_ (µg/mL) ± SD		
	*P. f.* (W2)	WI-26-VA4	SI
Jucá hydroalcoholic extract	11.10 ± 1.13	>100	9
Chloroquine	0.21 ± 0.13	>100	476
Artemether	0.01 ± 0.28	>100	10000

IC_50_: average inhibitory concentration; SD: standard deviation; *P. f.* (W2): chloroquine-resistant *P. falciparum*; WI-26-VA4: human cell fibroblasts; SI: selectivity index.

## Data Availability

The raw data from this work are available upon request to the corresponding author.
